# Dew and frost do not serve as water sources for rock-dwelling organisms in the Dry Valleys of Antarctica

**DOI:** 10.1007/s00425-026-05011-0

**Published:** 2026-04-29

**Authors:** Giora J. Kidron, Daniel Beysens, Christopher P. McKay

**Affiliations:** 1 Institute of Earth Sciences, The Hebrew University, 91904 Jerusalem, Israel; 2 OPUR, 2 rue Verderet, 75016 Paris, France; 3 Physique et Mécanique des Milieux Hétérogènes, CNRS, ESPCI Paris—PSL Université, Sorbonne Université, Sorbonne Paris Cité, 10 rue Vauquelin, 75005 Paris, France; 4 Space Science Division, Ames Research Center, National Aeronautics and Space Administration, Mountain View, CA USA

**Keywords:** Condensation, Lichens, Lithobionts, Non-rainfall water (NRW), Snowmelt, Sublimation

## Abstract

**Main conclusions:**

Contrary to previous assumptions, dew and frost cannot be regarded as water sources for lithobionts in the McMurdo Dry Valleys (MDV) of Antarctica, which therefore solely rely on snowmelt water for growth. Therefore, and contrary to previous claims, MDV can be regarded as a good analogue for life on Mars.

**Abstract:**

Rock-dwelling chlorolichens and cyanobacteria provide most of the total biomass of the ice-free zone of Antarctica, i.e., the McMurdo Dry Valleys (MDV), and yet, the water sources of MDV are not clear. In addition to snowmelt water that provides water to the lichen-dominated cryptoendolithic communities, many scholars also advocate the use of dew or frost as important water sources for chasmoendolithic cyanobacteria. The implications of these suggestions are large, especially due to the fact that the MDV serves as an important analogue for life on Mars. Based on 5-year long analysis of the four growing months (November, December, January, February) in three stations, we show that the likelihood of both sources to take place is low. Rock temperatures as measured by one of the stations (Marble Point) allowed us to also perform a detailed analysis. Rock temperatures never reached the dewpoint temperature, excluding the formation of dew. As for frost, the likelihood is extremely low during the growing season and an optimistic evaluation yields a possible occurrence of frost for 0.8 h per year. We, therefore, conclude that neither dew nor frost may serve as a meaningful water source for the chasmoendolithic cyanobacteria, which, therefore, rely on snowmelt water only. The endolithic communities of the MDV may be justifiably regarded as the best analogue for life on Mars.

## Introduction

Photoautotrophic rock-dwelling organisms/microorganisms (lithobionts), such as lichens and cyanobacteria are ubiquitous in all continents (Yu et al. 2020). This also includes the major part of the ice-free areas of Antarctica, i.e., the McMurdo Dry Valleys (MDV), where endolithic communities, i.e., organisms or microorganisms, whether cryptoendoliths (such as cyanobacteria or lichens residing within the rock pores) or chasmoendoliths (residing within rock fissures) inhabit the subsurface of rocks while being rare or absent on the rock surfaces (Friedmann 1982). With these endolithic communities yielding the highest biomass among all domains (Whittaker and Likens 1973; Kappen 2000), and following recent research aiming to detect possible extraterrestrial life, whether on Mars or other planets (eg. Davila and Schulze-Makuch 2016), these endolithic microorganisms have become a focus of extensive research. Considered by many scholars as an important analogue for Martian conditions (Pérez-Ortega et al. 2012; Zucconi et al. 2016), special attention is specifically given to their water sources in MDV.

Snowmelt water was long considered as the sole water source of these communities. Following snowmelt, water may infiltrate through the rock fissures and pores into the subsurface (~1 cm depth), where it may be protected from evaporation and serve as a water source for the endolithic communities (Friedmann 1982; Sun 2013). The use of snowmelt water by lichen-dominated cryptoendolithic communities was indeed substantiated following field observation (Sun 2013), and measurements of moisture (Kappen et al. 1981), relative humidity (RH) (Friedmann et al. 1987) and photosynthesis (Kappen and Friedmann 1983; Friedmann et al. 1993). According to Kappen et al. (1981), snowmelt water may infiltrate to about 10 mm, where it may be retained for more than several days. Subsequently, in comparison with a dry weight of 0.005–0.03% in the upper rock layer, i.e., the weight of water within the upper rock layer under dry conditions, these authors reported moisture of 0.05–0.12% 5 days after a snowfall. Following a snow event, moisture at the upper 10 mm layer also resulted in high RH (≥ 80%) at the rock pores lasting for several days (Kappen et al. 1981), and up to 2 weeks (Friedmann et al. 1987), which may thus provide sufficient moisture for net photosynthesis for cyanobacteria (through liquid water) and lichens (also through high RH). All the above measurements were conducted at the high elevations of MDV (>1000 m amsl), where above-freezing air temperatures do not take place, but the rocks may be warmed by summer sunlight to over 10 °C (Friedmann et al. 1987).

However, Büdel et al. (2008, 2025) suggested that dew and frost (which the authors inaccurately described as rime) may also serve as important water sources for chasmoendolithic cyanobacteria, which inhabit rock surfaces at the low elevations (< 1000 m amsl) of MDV. Although their research was criticized as not ‘well quantified’ (*sensu* Convey et al. 2014), the possible use of dew/frost was considered as a likely water source by numerous scholars (e.g., Quesada and Vincent 2012; Sterflinger et al. 2012; Wierzchos et al. 2012; Colesie et al. 2014; de los Rios et al. 2014; Makhalanyane et al. 2015; Pointing et al. 2015; McHugh et al. 2015; Crits-Christoph et al. 2016; Raggio et al. 2016; Meslier and DiRuggiero 2019; Bay et al. 2021). The notion that microorganisms at the low elevations of the MDV also benefit from dew/frost was, therefore, considered. If dew serves as regular water supply for the MDV communities, it was suggested that the MDV can no longer be considered as residing at ‘the edge of life’ (Büdel et al. 2008) and, therefore, should not be regarded as a good analogue for the Martian conditions. While the reasons outlined by Büdel et al (2008) were recently thoroughly refuted (Kidron et al. 2025), mainly due to errors in the temperature measurements and calculations, a systematic analysis of the climatological conditions of the low elevation parts of MDV is still required.

For vapor condensation, the substrate temperature (*T*_*s*_) should be equal or below the dewpoint temperature (*T*_*d*_), i.e., *T*_*s*_ ≤ *T*_*d*_. Similarly, frost will take place once the frostpoint temperature (*T*_*f*_) is reached, i.e., when *T*_*s*_ ≤ *T*_*f*_. Because the ice saturation pressure is slightly lower than the liquid water saturation pressure (by up to 25% depending on temperature), *T*_*f*_ is slightly lower than *T*_*d*_, i.e., *T*_*f*_ < *T*_*d*_. Theoretically, by providing liquid water, dew and frost may also facilitate net photosynthesis to lithobionts in MDV. Nevertheless, ice sublimation may drastically limit the presence of liquid water (Hagedorn et al. 2007; Levy 2021), and to the best of our knowledge, no accounts were yet published reporting on the visible occurrence of dew or frost during the summer growing season at any elevation in MDV.

Hypothesizing that the conditions in MDV are too dry to result in meaningful non-rainfall water (NRW), and therefore, the disparity between the populations in MDV (chasmoendoliths, cryptoendoliths) is not due to different water sources (whether dew/frost or rain/snow), analysis of the conditions conducive for NRW were carried out. To this end (i) we analyzed the main meteorological variables measured in meteorological (MET) stations, i.e., air temperature (*T*_*a*_), RH, photosynthetic active radiation (PAR) (i.e., the solar radiation wavelengths which can be used by photoautotrophs, such as cyanobacteria, alga, lichens and plants for photosynthesis), wind speed (WS) and direction (WD), and (ii) we calculated *T*_*d*_ and *T*_*f*_. We focused on data from low-elevation valley floors, where dew formation is most likely to occur. The Lake Fryxell, the Explorer Cove and the Marble Point stations were used to evaluate the potentiality for dew and frost based on the air temperatures (*T*_*a*_), whereas rock temperatures that were available from the Marble Point MET station served to estimate the time during which dew and frost are likely to take place. Analysis used data collected between 2015 and 2020.

## Materials and methods

### General and site description

Occupying 0.34% (4800 km^2^) of the entire continent (Convey et al. 2014), and constituting the largest ice-free zone in Antarctica, MDV is characterized by steep and high mountains (up to 2000 m-high) divided by narrow valleys. The climate is dry and cold (Doran et al. 2002; Obryk et al. 2020), with annual precipitation (in the form of snow) ranging from 3 to 50 mm water equivalent (Fountain et al. 2010). Average annual temperatures and relative humidity (RH) are −20 °C and 65%, respectively, while being higher and lower, respectively, during the growing season (Obryk et al. 2020). While air temperatures may seldom rise to ~10 °C at low altitudes during the austral summer months (November–February), which are characterized by continuous sunlight, they do not rise above the melting point (0 °C) at high (> 1 km) elevation (McKay 2015; Obryk et al. 2020). High velocity downslope winds (katabatic winds) that may reach speeds of up to 49 m s^−1^ (Obryk et al. 2020) add to the dryness and the harsh conditions of MDV (Clow et al. 1988). Due to the above constraints, possible growth conditions are confined to the summer months, between November and February, during which air temperatures may be warm enough, i.e., > −5 °C (Meyer et al. 1988) to allow for microorganism activity.

MDV is barren of vascular plants (Virginia and Wall 1999), and despite episodic moistening that can be noted by slight soil darkening (Levy 2013), they lack biocrusts (Pointing et al. 2009; Sterflinger et al. 2012). Epilithic lichens are extremely scarce and confined to very small and protected niches, while chasmoendolithic cyanobacteria were found to inhabit 30–80% of all available granitic substrates (Yung et al. 2014). And thus, in addition to photoautotrophs within lakes, endoliths constitute most of the photoautotrophs in MDV, and in fact, yield the highest biomass among all domains (Whittaker and Likens 1973; Kappen 2000). Due to their harsh growth conditions, these lithobionts are commonly considered as the most relevant Mars growth forms on Earth (Pérez-Ortega et al. 2012; Zucconi et al. 2016).

**Fig. 1:**
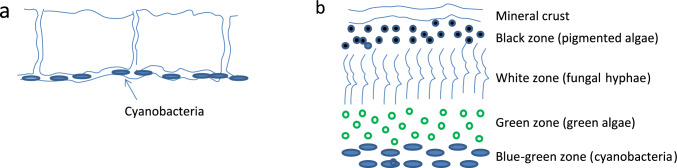
Schematic description of the chasmoendolithic (**a**) and cryptoendolithic (**b**) communities. Whereas the chasmoendoliths resides under and between fissures, the cryptoendoliths resides within the rock pores

Among the endolithic communities, chasmoendolithic cyanobacteria (CEB) and lichen-dominated cryptoendolithic communities (CEC), which mainly inhabit granite and sandstones, respectively, predominate (Fig. 1). As for the CEB, they are predominated by *Chroococcidiopsis* sp., and can be found in relatively wind-protected rock surfaces, where they dwell in horizontal fissures up to 8 mm in depth (Büdel et al. 2008). As for the CEC community, it inhabits the Beacon Sandstone formation which is found >1000–1200 m-high mountains (Friedman 1982). The CEC community is characterized by a unique ~10 mm-thick structure, consisting of ~1 mm-thick silicified mineral layer that covers the rock surface and 3–4 layers of microorganisms: (i) a top layer with a blackish–greenish color (consisting of green algae and pigmented fungi), (ii) a layer with non-pigmented white fungal hyphae, and (iii) a layer with green algae (*Hemichloris antarctica*) (Tschermak-Woess and Friedmann 1984), and (iv) occasionally, a blue–green layer of cyanobacteria (which, as defined by de la Torre et al. (2003) predominated by *Plectonema* sp.) is present at the very bottom of this lithic layer. According to de la Torre et al. (2003) the green algae and the fungi form a lichen, similar to an epilithic lichen *Buellia* sp. but with different cellular morphologies. Inhabiting the subsurface of rocks and boulders, CEC and CEB are subjected to sufficient PAR, but are relatively protected from ultraviolet radiation, UV (which is by 50–130% higher than in common temperate zones; Coleine et al. 2020), and the harmful effect of the high-velocity winds that characterize the MDV. Residing at the subsurface, CEB and CEC are also thought to benefit from many hours of wetness duration (Kappen et al. 1981).

Both lithobionts have different requirements for water in accordance with their domain (prokaryotes, eukaryote). While cyanobacteria (prokaryotes) require liquid water for net photosynthesis (with 0.1 mm marking the threshold; Lange et al. 1992), eukaryotes, such as green algae or chlorolichens (lichens with green algae as photobionts) may also utilize high RH of 70% and 80% marking the thresholds for respiration and net photosynthesis, respectively (Lange et al. 1986; Kappen et al. 1979). By attaching cloths and fragments of the lichen thali of *R. maciformis,* both of the same dimension (~2.5 × 1.5 cm) to different rock surfaces in the Negev (Kidron et al. 2014), a comparison of the amount of water condensed on both substrates was feasible. The water thresholds for respiration at RH=70%, which was found by Kappen et al. (1979) to correspond to 14% of the dry mass of *R. maciformis,* was found to equal 13.7% of the cloth, while RH=80%, which was found by Kappen et al. (1979) to be equivalent to 20% of the dry mass of *R. maciformis,* was found to be equal to 18.5% of the cloth. When divided by the surface area, these volumetric water content corresponded to 0.03 (70%) and 0.05 mm (80%) (Kidron and Starinsky 2019). These values correspond to the threshold values reported by Lange et al. (1970) for the crustose lichens that abound in the Negev. Clearly, any attempt to evaluate the growth conditions of the CEB and CEC should be similarly linked to their growth requirements. Specifically, the growth requirements of the CEB and CEC may help to determine whether or not photosynthesis that was recorded by Büdel et al (2008) in the CEB habitat, could have resulted from dew formation.

**Fig. 2:**
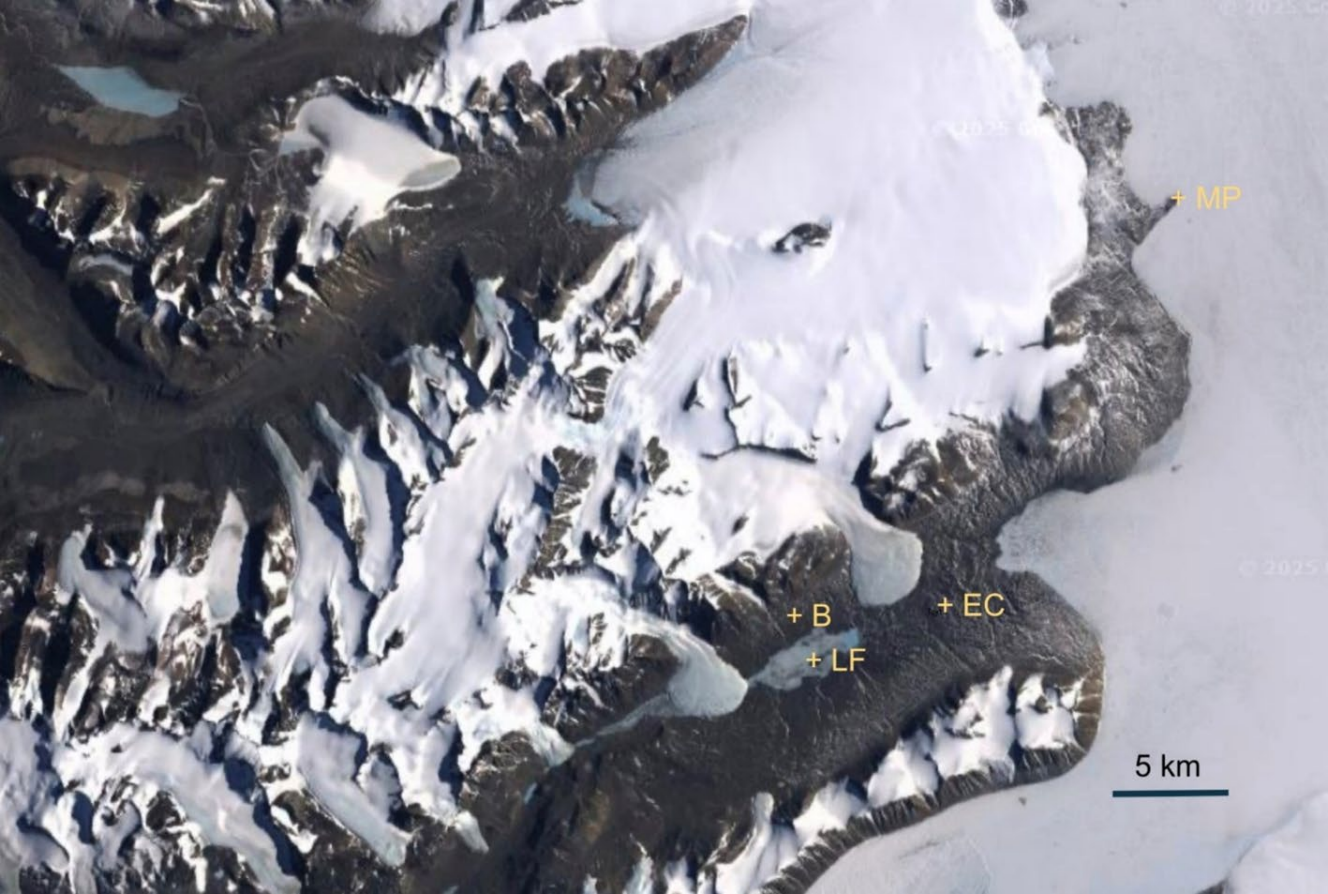
Location of all three MET stations, Lake Fryxell (LF), Explorer Cove (EC) and Marble Point (MP). The site investigated by Büdel et al. (2008) is marked as B. Note the proximity of Lake Fryxell and Explorer Cove to the Büdel site (all within the MDV inland) and the coastal location of the Marble Point

For the evaluation of the conditions which may allow for dew/frost formation, basic climatic conditions, as measured at Lake Fryxell (Doran and Fountain 2023a), the Explorer Cove (Doran and Fountain 2023b) and Marble Point (Seybold et al. 2009) MET stations were analyzed (Fig. 2). The Lake Fryxell station is in Taylor Valley on a small island (76°33’48"S, 163°29’17"E) in the southern part of the lake at an elevation of 25 m amsl. The Explorer Cove station is on a small ridge in Taylor Valley near the shore of McMurdo Sound (77°35’19.3"S 163°25’03.0"E) at an elevation of 79 m amsl. As for Marble Point **(**77°26′00″S, 163°50′00″E), the station lies on the shore of the Ross Sea, 21 m amsl, ~20 km from the coast, and ~20 km Northeast of Explorer Cove.

**Fig. 3:**
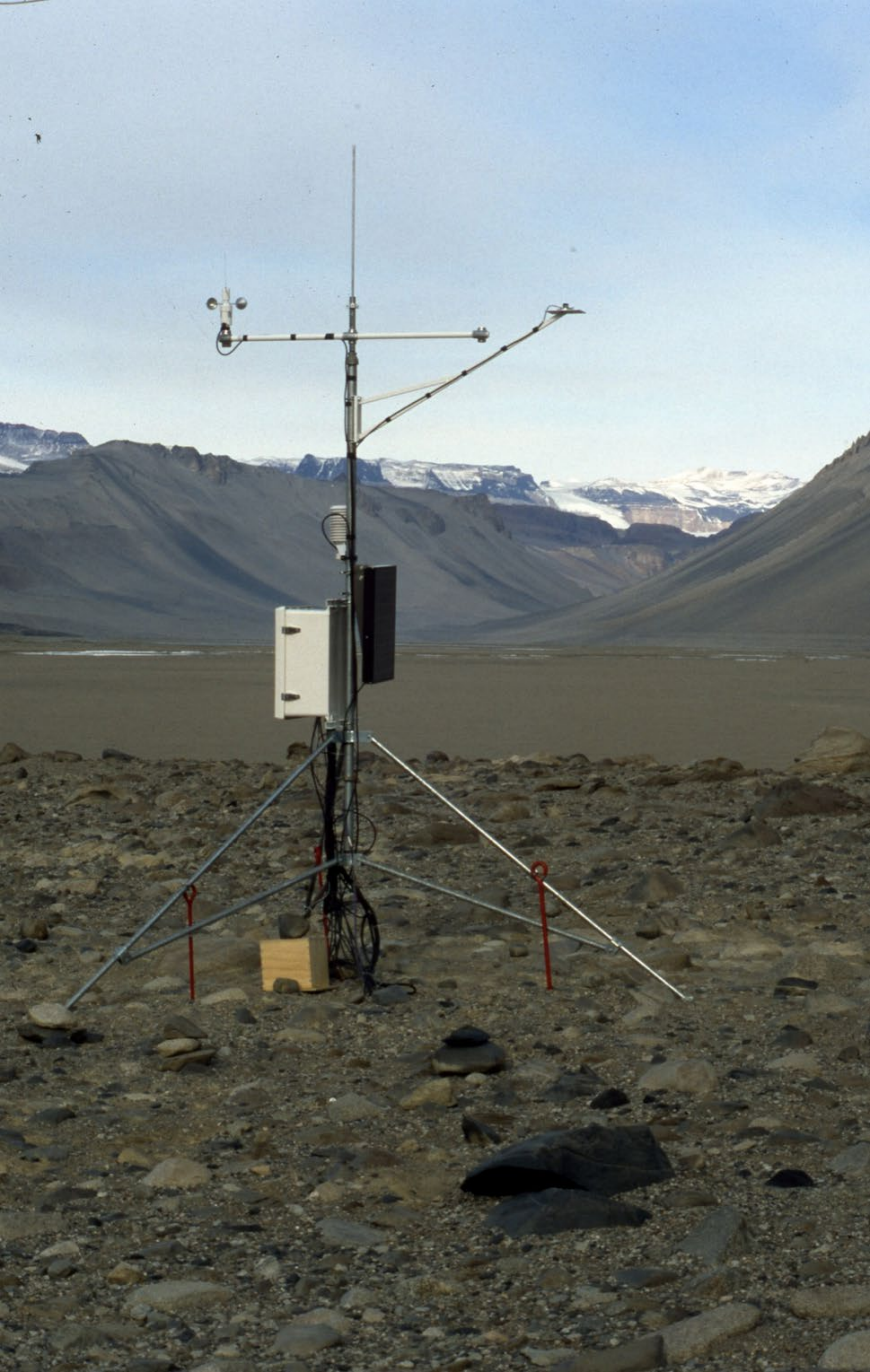
Met station at Taylor Valley showing typical granite stones and boulders inhabited by chasmoendolithic cyanobacteria (photograph by Ian Campbell)

These stations represent extensive year-round meteorological datasets for the lower elevations of the Dry Valleys, where stones and boulders with CEB characterize the valley floor (Fig. 3). These stations facilitate us the calculation of the conditions for dew and frost, with the Marble Point station also including rock surface temperatures. For the rock surface temperatures, Campbell-107 thermistor sensors, which was attached to the rock and shielded from direct radiation, were used.

### Meteorological measurements

Analyses were carried out based on the summertime data of 2015–2020 (November–February, except for November and December of 2015 in Explorer Cove due to missing data). In this period, the temperatures are relatively mild and radiation is sufficiently high to allow for possible photosynthesis. Our analysis included calculations of the average *T*_*a*_, RH, PAR, WS, WD, *T*_*d*_ and *T*_*f*_, and calculation of the likelihood of dew and frost to form on the rock surfaces, based on the rock temperature data available at the Marble Point MET station.

Air temperature and relative humidity at the Lake Fryxell and Explorer Cove stations were collected at 3 m-height above ground with a Campbell 207 probe (Obryx et al. 2020). At the Marble Point site, for the air temperature data, Campbell type 107 PRT sensors (2 m-high). RH was measured with Vaisala HMP45C T/RH probe at 1.6 m, PAR was measured (at 1.6 m) with LiCor 200x pyranometer, while for the wind speed and direction, Bendix Aerovane Model 120 sensors, installed at 3 m above ground, were used.

### Quantification of dew/frost amounts

Dew/frost is commonly calculated based on standard FAO equations during which the saturated and the actual vapor pressures are first calculated (Allen et al. 1998):

1 $$e_{s} = \, 0.6108\,\exp \left( {{{17.277T_{a} } \mathord{\left/ {\vphantom {{17.277T_{a} } {\left( {237.3 + T_{a} } \right)}}} \right. \kern-0pt} {\left( {237.3 + T_{a} } \right)}}} \right)$$

where *e*_*s*_(kPa) is the saturation vapor pressure at air temperature *T*_*a*_ (°C).

The actual vapor pressure is then calculated:

2 $$e_{a} = \, {{e_{s} \times RH} \mathord{\left/ {\vphantom {{e_{s} \times RH} {100}}} \right. \kern-0pt} {100}}$$

where *e*_*a*_ is the actual vapor pressure (kPa) and RH is the relative humidity (%).

However, as reported by Anderson (1994), RH reading at <0 °C should be corrected to another value, RH_i,_ when frost is present, due to the lower ice saturation pressure *e*_*si*_:

3 $$RH_{i} = {{e_{a} \left( {T_{a} } \right)} \mathord{\left/ {\vphantom {{e_{a} \left( {T_{a} } \right)} {e_{si} \left( {T_{a} } \right)}}} \right. \kern-0pt} {e_{si} \left( {T_{a} } \right)}}$$

One notes that RH_i_ > RH. The real RH, i.e., RH which will allow for icing (RH_i_) may be by approximately 10% higher at −10 °C and by 20% higher at < −20 °C. It can be calculated from the ratio *e*_*si*_/*e*_*a*_ according to the equation:

4 $$RH_{i} = \left( {1 - 0.01 * T_{a} } \right) * RH_{meas}$$

where RH_i_ and RH_meas_ imply corrected and measured RH, respectively. One notes that this equation is a linear approximation of the ratio *e*_*si*_/*e*_*a*_. RH_i_ values slightly larger than 100% can be sometimes obtained. These values correspond to supersaturated air with respect to the ice saturation pressure and the possibility to form snow on nucleation sites (see also Anderson 1994).

The dewpoint temperature (*T*_*d*_) is thereafter calculated from the inversion of Eq. (1):

5 $$T_{d} = \left( {{{{116}.{91} + {237}.{\mathrm{3ln}}\left( {e_{a} } \right)} \mathord{\left/ {\vphantom {{{116}.{91} + {237}.{\mathrm{3ln}}\left( {e_{a} } \right)} {\left( {{16}.{78}{-}{\mathrm{ln}}\left( {e_{a} } \right)} \right)}}} \right. \kern-0pt} {\left( {{16}.{78}{-}{\mathrm{ln}}\left( {e_{a} } \right)} \right)}}} \right)$$

While the frostpoint, which is slightly below the dewpoint, is calculated according to the following equation (Iribarne and Godson 1974):

6 $$T_{f} = T_{d} + \left( {{{2671.02} \mathord{\left/ {\vphantom {{2671.02} A}} \right. \kern-0pt} A}} \right) - T_{a}$$

7 $${\mathrm{With}}\,A = \left( {{{2954.61} \mathord{\left/ {\vphantom {{2954.61} {T_{a} }}} \right. \kern-0pt} {T_{a} }}} \right) + \left( {2.193665*\ln \left( {T_{a} } \right)} \right) - 13.3448$$

Our calculations thus involved several stages:

- Marking all above-freezing temperatures, i.e., *T*_*a*_ > 0 ℃; calculating *T*_*d*_ and then the time during which *T*_*a*_ ≤ *T*_*d*_ and the time during which *T*_*rock*_ ≤ *T*_*d*_ (for Marble Point). Note that except for very rare occasions of warm winds, *T*_*rock*_ was always higher than *T*_*a*_.
- Marking all negative temperatures between 0 ℃ and −5 ℃, i.e., during which liquid water may still be available for lithobionts (Meyer et al. 1988). We calculated RHi and then *T*_*f*_ and the time during which *T*_*a*_ ≤ *T*_*f*_ and the time during which *T*_*rock*_ ≤ *T*_*f.*_
- To exclude, as much as possible, hours during which the substrate temperatures are lower than *T*_*f*_ due to snowfall, we separately marked all times during which *T*_*rock*_ ≤ *T*_*f.*_ occurred during ‘daylight’. We considered ‘daylight’ in between 08:00 and 18:00 while considering ‘nighttime’ hours during which non-rainfall water (NRW), i.e., dew, fog or vapor at high RH may accumulate between 18:00 and 08:00. In addition, we excluded all times during which *T*_*a*_ or *T*_*rock*_ took place under wind speed ≥ 4.5 m s^−1^, which would hinder dew/frost formation (Monteith 1957; Beysens et al. 2006; Muselli et al. 2009).
- Since snow events are characterized by RH ≥ 95%, by a reduction in PAR (Bruland and Hagen 2002) and may also take place during daytime (08:00–18:00), we assumed that such conditions indicate snow events.
- Since dew forms on top of the surface while CEB dwell endolithically, the time duration during which *T*_*rock*_ or *T*_*a*_ reach *T*_*d*_ or *T*_*f*_ has to be long enough to result in sufficient liquid water. Taking maximum condensation rate of 0.025–0.035 mm h^−1^ (Monteith 1957; Kidron and Starinsky 2019), it implies >3 h of consecutive conditions during which *T*_*rock*_ ≤ *T*_*d*_ or *T*_*rock*_ ≤ *T*_*f*_ . Events during the night during which these conditions were met were marked and their time duration was calculated.

## Results

With the aim to better reflect the climatic conditions of the inland MDV (rather than the coast), and as close as possible to the Büdel site, specific meteorological data from the Lake Fryxell MET station were analyzed, followed by an analysis of all three MET stations.

**Fig. 4:**
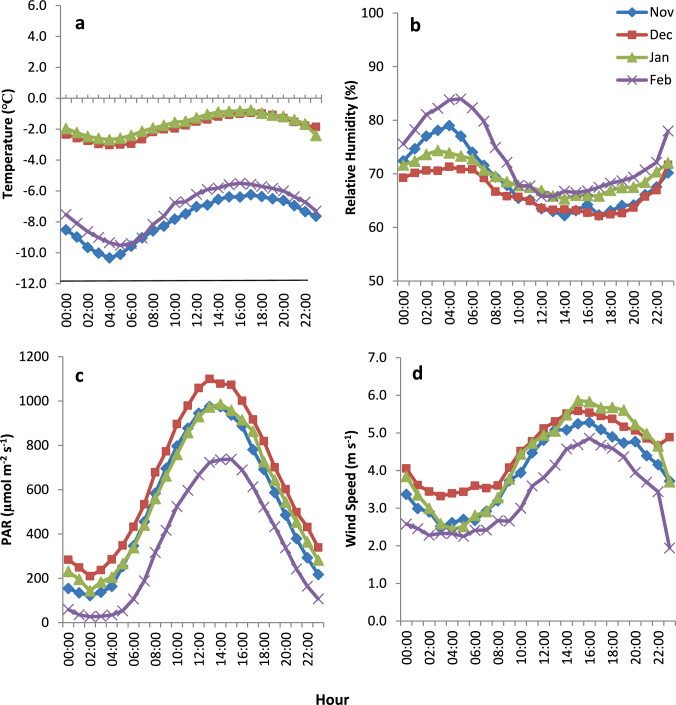
Average hourly values of temperatures (**a**), relative humidity (**b**), PAR (**c**) and wind speed (**d**) during the months November, December, January and February as calculated for the Lake Fryxell MET station during 2015–2020. Bars represent one standard error (*n*= 14309)

The typical daily (diel) values of temperature, RH, PAR and WS for November, December, January and February, as calculated for 2015–2020 for the Lake Fryxell MET station are shown in Fig. 4. One can note that large differences exist between the mid austral summer (December, January) and the remaining summer months (November, February). While air temperatures between −1 ℃ and −3 ℃ characterize the mid-summer months, air temperatures of −5 ℃ to −10 ℃ characterized the months of November and February (Fig. 4a), which may not be conducive for lithobiont growth. Notably, however, are the lower maximum nighttime RH values (60–75%) that characterize the mid-summer months, which are thus less conducive for condensation in comparison with November and February (65–85%) (Fig. 4b). As for PAR (Fig. 4c) and WS (Fig. 4d), November was characterized by a relatively different pattern with substantially lower PAR during nighttime and substantially lower WS during the afternoon. WD was similar during all months (not shown).

Table 1 summarizes the climatic variables for Lake Fryxell, Explorer Cove and Marble Point. Overall, all stations exhibited similar values, except for the wind direction that differed in Marble Point (~ 200°) in comparison with Lake Fryxell and Explorer Cove (~ 85°), apparently reflecting the channeling effect of Taylor Valley on Explorer Cove and Lake Fryxell compared to the Marble Point station 20 km away from that Valley and on the coast. As far as the above-freezing temperatures are concerned, they characterize 14.1% of all temperatures in Lake Fryxell while only 7.3% in Explorer Cove and Marble Point, respectively. However, as far as the *T*_*d*_ is concerned, it was higher in Marble Point (−7.8 ℃) in comparison with Lake Fryxell (−8.6 ℃) and Explorer Cove (−10.2 ℃). Similarly, as far as *T*_*f*_ is concerned, it was similar in Marble Point and Lake Fryxell (−7.7 ℃), with both stations yielding lower temperatures in comparison with Explorer Cove (−6.5 ℃). In this regard, one should note that *T*_*a*_–*T*_*d*_ was lower for Marble Point (8.9 ℃) in comparison with Lake Fryxell (10.1 ℃) and Explorer Cove (11.6 ℃), whereas *T*_*a*_–*T*_*f*_ was slightly higher for Marble Point (−5.1 ℃) in comparison with Lake Fryxell (−5.3 ℃) and Explorer Cove (−5.6 ℃), reflecting slightly milder climate conditions at Marble Point. As for the number of hours during which *T*_*a*_–*T*_*f*_ (for air temperatures between 0 ℃ and −5 ℃), the values were similar 47.7% and 45.4% for Lake Fryxell and Explorer Cove, respectively, in comparison with 48.2% in Marble Point.

**Table 1:** Average hourly values for 2015-2020 during the entire year (a), during days when *T*_*a*_ > 0 (℃) (b) and during days when −5 <* T*_*a*_ < 0 (℃) (c) for Lake Fryxell, Explorer Cove and Marble Point

Variable	Lake Fryxell (*n*= 14309)	Explorer Cove* (*n*= 13058)	Marble Point (*n*= 14408)
a. All			
*T*_*a*_ (℃)	−4.5 (4.7)	−5.5 (4.5)	−5.3 (4.4)
PAR (µmol m^−2^ s^−1^)	534.9 (372.1)	400.1 (294.3)	460.8 (377.0)
WS (m s^−1^)	4.1 (2.5)	3.4 (2.1)	3.4 (2.0)
WD (º)	85.1 (78.3)	85.5 (79.9)	201.1 (83.0)

Variable	Lake Fryxell (*n*= 2020)	Explorer Cove* (*n*= 956)	Marble Point (*n*= 1035)
b. *T*_*a*_ > 0 (℃)			
*T*_*a*_ (℃)	1.6 (1.3)	1.4 (1.4)	1.1 (1.0)
RH (%)	48.8 (14.6)	44.6 (16.4)	52.9 (13.3)
PAR (µmol m^−2^ s^−1^)	840.5 (334.9)	583.9 (283.0)	836.6 (358.4)
WS (m s^−1^)	5.3 (2.5)	4.0 (2.5)	3.4 (2.4)
WD (º)	82.4 (74.2)	83.0 (76.1)	204.4 (91.8)
*T*_*d*_ (℃)	−8.6 (3.6)	−10.2 (4.4)	−7.8 (3.1)
*T*_*a*_–*T*_*d*_ (℃)	10.1 (4.2)	11.8 (5.1)	8.9 (3.4)
T_a_ > 0 (h yr^−1^) (Ave; SD; %)	404.0; 139.4; 14.1%	191.2; 79.2; 7.3%	207.0; 127.6; 7.3%

Variable	Lake Fryxell (*n*= 6841)	Explorer Cove* (*n*= 5923)	Marble Point (*n*= 6969)
c. −5 <* T*_*a*_ ≤ 0 (℃)			
*T*_*a*_ (℃)	−2.4 (1.4)	−2.7 (1.4)	−2.6 (1.4)
RH_i_ (%)	67.4 (16.4)	66.1 (17.6)	66.0 (13.0)
PAR (µmol m^−2^ s^−1^)	569.6 (357.7)	452.1 (289.7)	605.2 (366.8)
WS (m s^−1^)	4.4 (2.4)	3.3 (2.1)	3.3 (1.9)
WD (º)	76.4 (71.6)	66.2 (70.6)	194.9 (91.2)
*T*_*f*_ (℃)	−7.7 (3.4)	−6.5 (4.1)	−7.7 (2.8)
*T*_*a*_ - *T*_*f*_	−5.3 (3.5)	−5.6 (4.4)	−5.1 (2.9)
−5 ℃ <* T*_*a*_ ≤ 0 ℃ (h yr^−1^) (Ave; SD; %)	1368.3; 168.7;47.7%	4738.4; 1441.8;45.4%	1393.8; 205.7;48.2%

^*^Calculations do not include the months November and December, 2015 due to missing data

One standard deviation in parenthesis. *T*_*a*_ air temperature; *T*_*d*_ dewpoint temperature; *T*_*f*_ frostpoint temperature; *RH* relative humidity; *RH*_*i*_ corrected relative humidity; *PAR* photosynthetic active radiation; *WS* wind speed; *WD* wind direction

The potential for dew and frost formation on the rock surfaces is shown for Marble Point, where rock temperatures were measured. Although warm air spells occurred during which air temperatures were higher than the rock temperatures, this took place only on seldom occasions. Almost always rock temperatures were higher than air temperatures. This was especially the case for the minimum temperatures during the warmest month, January. When the minimum temperatures at 06:00 were calculated for *T*_*a*_ and *T*_*rock*_, they were on average 4.3 ℃ warmer at the rock surfaces (Table 2). The higher rock temperatures were also reflected by the higher temperature differences between *T*_*rock*_ and *T*_*d*_ (14.4 ℃) in comparison with *T*_*a*_–*T*_*d*_ (8.9 ℃) (Tables 1 and 3). A similar trend also characterized the below-zero temperatures, with *T*_*a*_ –*T*_*f*_ and *T*_*rock*_ –*T*_*f*_ yielding −5.1 ℃ and −8.3 ℃, respectively (Tables 1 and 3), also pointing to the higher temperature difference between *T*_*rock*_ and *T*_*f*_ in comparison with *T*_*a*_ and *T*_*f*_.

**Table 2:** Comparison between the minimum air and the rock temperatures (calculated daily) during the month of January at Marble Point. One standard deviation in parenthesis (*n*=31)

Year	Air	Rock
2015/16	−1.7 (2.6)	2.4 (2.2)
2016/17	−3.0 (1.7)	1.0 (1.3)
2017/18	−3.1 (1.9)	0.6 (1.7)
2018/19	−3.1 (1.8)	1.7 (2.4)
2019/20	−3.4 (2.1)	1.6 (1.9)
Total	−2.8 (0.7)	1.5 (0.7)

**Table 3:** Relative humidity and annual duration (h yr^−1^) for 2015–2020 during which (a) dew (*n*= 1035) and (b) frost (*n*= 6969) may take place on the rock surface at Marble Point

Ta > 0 °C	
* T*_*a*_–*T*_*d*_ (°C)	9.0 (3.4)
RH (%)	52.4 (13.4)
T_rock_–*T*_*d*_ (°C)	14.4 (4.7)
T_rock_ < *T*_*d*_ (h yr^−1^)	0
0 °C > Ta > −5 °C	
T_rock_ < *T*_*f*_ (°C)	−8.3 (4.9)
T_rock_ < *T*_*f*_ (h yr^−1^)	49.4 (54.0)
RH_i_ (%)	67.8 (14.2)
T_rock_ < *T*_*f*_ while wind speed < 4.5 m s^−1^ (h yr^−1^)	40.6 (45.3)
T_rock_ < *T*_*f*_ during the night (20:00 to 08:00) (h yr^−1^)	14.4 (19.0)
T_rock_ < *T*_*f*_ while lasting > 3 h (h yr^−1^)	9.2 (11.5)
T_rock_ < *T*_*f*_ when excluding apparent snow events (h yr^−1^)*	0.8 (1.8)

*When temperatures during which T_rock_ < *T*_*f*_ began already at noon or continued until noon the next day.

One standard deviation in parenthesis. *T*_*a*_ air temperature; *T*_*d*_ dewpoint temperature; *T*_*f*_ frostpoint temperature; *T*_*rock*_ rock temperature; *RH* relative humidity; *RH*_*i*_ corrected relative humidity

Table 3 calculates the average number of annual hours during which *T*_*rock*_ ≤ *T*_*f*_. When considering the number of hours per year (49.4 h), one realizes that only during a small fraction of this time frost may take place, since (i) part of the time (8.6 h) wind speed is higher than 4.5 m s^−1^, which do not facilitate dew or frost formation, (ii) part of the time (24.4 h) *T*_*rock*_ ≤ *T*_*f*_ occurred during daytime, while (iii) part of the time (5.0 h) consecutive hours during which frost may condense did not take place. Adding likely snow events, as indicated by events during which *T*_*rock*_ ≤ *T*_*f*_ began during the day or continued into the following noon time, the likelihood during which *T*_*rock*_ ≤ *T*_*f*_ takes place during the growing season is negligible, 0.8 h per year.

## Discussion

Lithobiont inhabitation of the rock surfaces of MDV on one hand, and the central role played by NRW in the growth and distribution of lithobionts in hot deserts on the other hand (Lange et al. 1970; Kidron et al. 2023), caused numerous scholars to suggest that NRW may also serve as an important water source for lithobionts in MDV, but as will be shown below, the meteorological conditions prevailing in MDV exclude such possibility.

This is supported by our analysis of the climatic variables. Average diel air temperatures show a distinct difference between the months of November and February during which temperature fluctuate between −5 ℃ and −11 ℃ (Fig. 4a), i.e., below the range of temperatures that may facilitate lithobiont growth, and the midsummer months (December, January), characterized by milder temperatures (commonly fluctuated between −1 ℃ and −3 ℃). Yet, while providing better growth conditions, midsummer months are characterized by lower RH, with average maximum RH of ~68% (Fig. 4b). As for PAR and WS, PAR is commonly sufficient for lithobiont growth through the entire summer months (generally above 100 µmol m^−2^ s^−1^ which is required for net photosynthesis; Lange et al. 1992). While WS is ≥ 4.5 m s^−1^ during the afternoon and early evening (found to prevent dew/frost formation; Monteith 1957; Beysens et al. 2006; Muselli et al. 2009), it is lower during nighttime (~ 2–3.5 m s^−1^) and thus should not hinder dew/frost formation during many of the nights.

As for dew, only 7.3–14.1% of the all temperatures exhibited *T*_*a*_ > 0 ℃ (Table 1b). Notwithstanding is the relatively low RH that characterize hours with *T*_*a*_ > 0 ℃, averaging between 44.6% and 52.9%, substantially lower than RH ≥ 82%, found as the minimum required value during which photoautotrophs may be activated (Kidron and Kronenfeld 2020). As for frost formation during temperatures between 0 ℃ and −5 ℃ (which still allow for liquid water availability and hence for possible lithobiont growth), relative humidity was still low, between 66% and 67% at all sites (Table 1c). With *T*_*rock*_ never reaching *T*_*d*_ in Marble Point, the option of dew was eliminated. A more thorough analysis is required, however, once frost is considered, i.e., during times during which *T*_*rock*_ ≤ *T*_*f*_.

Detailed daily analysis during which *T*_*f*_ –*T*_*rock*_ ≥ 0 ℃ as found for Marble Point during the growing period show very little likelihood also for frost formation (Table 3). Out of 49.4 h yr^−1^ during which *T*_*rock*_ ≤ *T*_*f*_ took place, part of these hours included also daytime hours or hours with strong winds during which frost would not form. In addition, taking the maximum condensation rate of 0.025–0.035 mm h^−1^ (Monteith 1957; Kidron and Starinsky 2019), it implies that in order for frost to form, >3 h of consecutive conditions during which *T*_*rock*_ ≤ *T*_*f*_ are necessary. Adding hours during which these conditions apparently reflected snow events (as supported by the fact that in certain occasions *T*_*rock*_ ≤ *T*_*f*_ began already during daytime or continued to the noon time the following day), the optimistic estimate for frost events during the growing season yields only 0.8 h per year, excluding the possibility that frost may serve as a meaningful water source for the lithic communities.

Whether dew or frost, the lower the value *T*_*a*_*–T*_*d*_ or *T*_*a*_–*T*_*f*_ for above- or below-freezing temperatures, respectively, the higher the likelihood for dew or frost formation, respectively (Beysens 2018). In all sites the differences were relatively large, implying very low likelihood for the formation of dew or frost (Table 1). Moreover, with the gradual accumulation of vapor that results in a gradual growth of the water droplets (Beysens 1995, 2006, 2018), any temperature fluctuations around 0 ℃ may also result in evaporation, hampering a continuous water accretion. With the threshold of liquid water of 0.1 mm required for cyanobacteria activity (Lange et al. 1992), and the minimum amount of 0.3 mm required for water trickling (Tomaszkiewicz et al. 2015), which may, therefore, allow the water to reach the chasmoendolithic habitat*,* the analyses of the meteorological data do not support the occurrence of dew or frost which may be available for CEB activity.

Supporting evidence for the absence of summer dew or frost is the amount of precipitable water which reflects the amount of the atmospheric vapor above a specific surface area. Precipitable water in Antarctica is among the lowest on Earth (Guillot et al. 2015), 3–4 times lower than that of the Negev (Tuller 1968), therefore, implying substantially lower opportunities for dew or fog formation. In addition, in comparison with soil (Levy 2021) or certain hygroscopic minerals (halite, gypsum) that readily adsorb vapor (Wierzchos et al. 2011), aeolian-derived salt is readily leached from the rocks and, therefore, cannot assist in vapor adsorption. One should note that the current calculations during which hours with *T*_*f*_–T_rock_ ≥ 0 ℃ were considered (Table 3) also apply to fog. All in all, NRW cannot be regarded as a meaningful water source for the MDV lithobionts, which in addition to water, also require adequate light and temperature conditions for growth.

The point made by various scholars advocating the retention of liquid water for days within the rock pores (McKay et al. 2024), and the advantage of sheltered locations and rough surfaces in retaining vapor or dew (Brutsaert 1975; Bohren 1988; 2006; Mott et al. 2016) is relevant. Brutsaert (1975) and Mott et al. (2016) discuss the advantage of surface roughness to facilitate vapor stagnation, supporting the advantage of the chasmoendolitic habitat. As observed by Friedmann et al. (1987) and Kappen et al. (1981) liquid water may be retained for long days within the pores and fissures which protect them from evaporation. Nevertheless, as emphasized by Bohren (1988, p. 30), this will take place only when “assuming a plentiful supply of water”, i.e., only when the substrate already contains sufficiently high amount of water.

Also, possible condensation within very tiny pores or fissures, termed ‘capillary condensation’ (Barsotti and Piri 2021) is not likely in non-hygroscopic minerals. The process, primarily described in shales, may take place below the saturation air pressure within the tiny pores (0.002–0.1μ) that allow for condensation even at RH< 100% (Benavente et al. 2009; Cihan et al. 2019), and predominantly at <0.01μ (Zhao and Yuan 2018). Yet, capillary condensations at RH<< 100% require pores of ~0.001μ, while it may take place at much larger pores of 0.05–0.1μ only at RH > 95% (Germinario et al. 2017). Granites are characterized by much larger pore sizes, with a typical median pore diameter of ~0.5μ (Gao et al. 2021). It is not only that such relatively large pore sizes are not conducive to capillary condensation, but that relative humidities greater than 95% are required; these are not common in the MDV. In this regard it is of interest to note that while samples of Navajo sandstone gained ~6% weight when wetted with liquid water, they only gained ~0.3% when exposed to 100% humidity (CP McKay, unpub.), reemphasizing the unlikelihood of high relative humidity to provide meaningful amount of water.

And not less important, even when taking the 0.05–0.1μ-pores that facilitate capillary condensation, these pores are one order of magnitude smaller than the cell diameter of *Chroococcidiopsis*, which range between 2μ and 6μ, and which, together with their thick sheath of EPS, reach 20–30μ (Büdel et al. 2008). This physical barrier coupled with the high RH required for capillary condensation under the typical diameter pores that characterize the granite would not facilitate cyanobacteria inhabitation (and subsequently activation). One may, therefore, assume that for the chasmoendoliths to be activated by dew, dew trickling should take place from the surface to the ~5 mm-depth, where the CEB reside. Nevertheless, dew trickling necessitates a minimum amount of 0.3 mm (Tomaszkiewicz et al. 2015), an amount that will be formed only after consecutive hours during which *T*_rock_ < *T*_*d*_—an unlikely occurrence in MDV.

We, therefore, suggest that similar to CEC, snowmelt serves as the water source for CEB. The occasionally relatively high temperatures of the rocks during daytime (up to 20 °C; Nienow et al. 1988) will allow for ice melting at the snow–rock interface (eg. Sun 2013). With both communities being sheltered (whether by the mineral seal above CEC or by the fissures within the granite rocks that host the CEB), snowmelt water may be relatively protected from rapid evaporation, providing growth conditions for both communities. In addition, as already observed and measured for CEC, the sealed habitat may retain the liquid water for 1–2 weeks after the actual wetting (Kappen et al. 1981; Friedmann et al. 1987). Similar conditions may also prevail at the CEB habitat, facilitating photosynthesis by the CEB as recorded by Büdel et al. (2008). Snowmelt water will allow for the activation of the cyanobacteria (by the liquid water) at CEB and CEC, and the activation of the green algae and chlorolichens (by the liquid water and also by the resultant high vapor content within the pores) at CEC.

The irregularity and scarce occasions of snowfall events may justify the common notion that the endolithic communities of the MDV may be justifiably regarded as the best analogue for life on Mars. In this respect, it is important to consider models that attempt to explain heat transfer from surfaces (Brutsaert 1975), and more recent models that offer convincing explanations for the existences of perennial snowfields (Williams et al. 2008; Mott et al. 2016) and the possible occurrence of liquid water withing snow/ice pores on Mars (Clow 1987; Christensen 2003; Khuller and Christensen 2021). These models brought scholars to suggest that extant life may be possible within snow pores (Khuller et al. 2024). Accordingly, thin films of water of ~ 0.3 mm may exist within the pores and given adequate temperatures and light (during the summer), possible life should not be excluded (Khuller et al. 2024). In this regard, one should note that in the heart of the Negev with ~ 95 mm of annual rain, < 100 h yr^−1^ of cyanobacteria activity were estimated to suffice cyanobacteria growth (Kidron 2026, Kidron et al. 2023). The presence of lithic cyanobacteria at the Negev fringes, where rainfall is lower (50–70 mm), suggest that several dozens of hours of activity may be sufficient for cyanobacterial establishment and growth.

## Conclusions

Daily analyses of the climatic conditions prevailing at three sites in the low elevations of the MDV, the Lake Fryxell, the Explorer Cove and the Marble Point do not support the view that lithobionts may benefit from dew or frost. Average RH at all stations during days with 0 ≤ *T*_*a*_ ≥ −5 ℃ was 66–67%, while being even lower (44–52%) during days during which *T*_*a*_ > 0 ℃, far below the RH values reported to facilitate vapor condensation. *T*_*rock*_ never reached *T*_*d*_ excluding the possible occurrence of dew formation on the rock surfaces. As for *T*_*f*_, our analysis shows that the likelihood of frost formation during the growth period is < 1 h a year, also disregarding frost as a likely water source for the lithic communities in MDV. Evidence suggests that moisture from snowmelt, as suggested for the CEC, is apparently the sole water source for CEB. Unlike dew or frost that may form on a regular basis, the MDV lithobionts benefit only from sporadic and low snow precipitation, and may be, therefore, considered as the best analog to Mars-like environments.

## Data Availability

All data used in this study was obtained from public sites for the LTER and NZ/USDA station.

## Abbreviations

CEB: Chasmoendolithic cyanobacteria
CEC: Cryptoendolithic community
e_a_: Actual vapor pressure
e_s_: Saturated vapor pressure
MDV: McMurdo Dry Valleys
NRW: Non-rainfall water
MET: Meteorological
PAR: Photosynthetic active radiation
RH: Relative humidity
RH_i_: Corrected RH
RH_meas_: Measured RH
Ta: Air temperature
Td: Dewpoint temperature
Tf: Frostpoint temperature
T_rock_: Rock temperature
WD: Wind direction
WS: Wind speed
